# The IDvIP Trial: A two-centre randomised double-blind controlled trial comparing intramuscular diamorphine and intramuscular pethidine for labour analgesia

**DOI:** 10.1186/1471-2393-11-51

**Published:** 2011-07-08

**Authors:** Michael YK Wee, Jenny P Tuckey, Peter Thomas, Sara Burnard

**Affiliations:** 1 Consultant Anaesthetist, Poole Hospital NHS Foundation Trust, Longfleet Road, Poole, BH15 2JB, England, United Kingdom. Visiting professor, Bournemouth University, Bournemouth BH1 3LT, England, UK; 2 Consultant Anaesthetist, Royal United Hospital, Combe Park, Bath BA1 3NG, England, UK; 3 Professor of Healthcare Statistics and Epidemiology, School of Health and Social Care, Bournemouth University, Bournemouth BH1 3LT, England, UK; 4 Research Co-ordinator, Royal United Hospital, Combe Park, Bath BA1 3NG, England, UK

## Abstract

**Background:**

Intramuscular pethidine is routinely used throughout the UK for labour analgesia. Studies have suggested that pethidine provides little pain relief in labour and has a number of side effects affecting mother and neonate. It can cause nausea, vomiting and dysphoria in mothers and can cause reduced fetal heart rate variability and accelerations. Neonatal effects include respiratory depression and impaired feeding. There are few large studies comparing the relative side effects and efficacy of different opioids in labour. A small trial comparing intramuscular pethidine with diamorphine, showed diamorphine to have some benefits over pethidine when used for labour analgesia but the study did not investigate the adverse effects of either opioid.

**Methods:**

The Intramuscular Diamorphine versus Intramuscular Pethidine (IDvIP) trial is a randomised double-blind two centre controlled trial comparing intramuscular diamorphine and pethidine regarding their analgesic efficacy in labour and their side effects in mother, fetus and neonate. Information about the trial will be provided to women in the antenatal period or in early labour. Consent and recruitment to the trial will be obtained when the mother requests opioid analgesia. The sample size requirement is 406 women with data on primary outcomes. The maternal primary outcomes are pain relief during the first 3 hours after trial analgesia and specifically pain relief after 60 minutes. The neonatal primary outcomes are need for resuscitation and Apgar Score <7 at 1 minute. The secondary outcomes are an additional measure of pain relief, maternal sedation, nausea and vomiting, maternal oxygen saturation, satisfaction with analgesia, whether method of analgesia would be used again, use of Entonox, umbilical arterial and venous pH, fetal heart rate, meconium staining, time from delivery to first breath, Apgar scores at 5 mins, naloxone requirement, transfer to neonatal intensive care unit, neonatal haemoglobin oxygen saturation at 30, 60, 90, and 120 mins after delivery, and neonatal sedation and feeding behaviour during first 2 hours.

**Discussion:**

If the trial demonstrates that diamorphine provides better analgesia with fewer side effects in mother and neonate this could lead to a change in national practice and result in diamorphine becoming the preferred intramuscular opioid for analgesia in labour.

**Trial Registration:**

ISRCTN14898678

Eudra No: 2006-003250-18, REC Reference No: 06/Q1702/95, MHRA Authorisation No: 1443/0001/001-0001, NIHR UKCRN reference 6895, RfPB grant PB-PG-0407-13170_IR5

## Background

Labour is generally considered to be a painful experience and analgesia is regularly required. Intramuscular opioids are one form of analgesia regularly employed. Intramuscular pethidine is routinely used throughout the UK for labour analgesia and is the only opioid licensed for independent use by midwives. There are a number of concerns in the literature regarding the use of pethidine. Some studies have suggested that pethidine provides little or no pain relief in labour, its main effect being to cause sedation rather than analgesia [1,2]. Pethidine also has a number of side effects on both the mother and the neonate which make it a less than ideal choice for labour analgesia. It can cause nausea, vomiting and dysphoria in women receiving it during labour [3]. It also crosses the placenta and can cause reduced fetal heart rate variability and fewer heart rate accelerations [4]. Effects on the neonate include respiratory depression, impaired feeding and altered crying [5-7].

Despite the disadvantages of pethidine, there are few large studies comparing the relative side effects and effectiveness of different opioids in labour and systematic reviews comparing parenteral opioids in labour have suggested a need for well-designed and suitably-sized trials of pethidine versus other opioids [8,9]. A small trial comparing intramuscular pethidine with diamorphine, showed diamorphine to have some benefits over pethidine when used for labour analgesia but the trial did not study the potential adverse effects of either opioid [10]. A national survey published in 2008 relating to use of intramuscular opioids for analgesia in labour in consultant-led units in the UK revealed that diamorphine was used in 34% of maternity units and was the most commonly used opioid for analgesia in labour in Scotland [11]. Where it is used, anecdotally it is perceived to provide superior analgesia with fewer side effects than pethidine, but to date there is no published large randomised controlled trial to support this impression. We aim to undertake a two-centre double-blind randomised controlled trial comparing intramuscular diamorphine and pethidine in terms of their analgesic efficacy in labour and their side effects in mother, fetus and neonate.

## Methods/design

This two-centre double-blind randomised controlled trial comparing intramuscular diamorphine and pethidine will be conducted at Poole Hospital NHS Foundation Trust (PHFT), the Sponsor site, and the Royal United Hospital, Bath (RUH). Southampton and South West Hampshire Ethics Committee granted ethical approval. Information about the trial will be provided to women and consent obtained in the antenatal period via the antenatal clinics, both in the community and maternity hospital, or in early labour in the delivery suite prior to the request for analgesia. Consented women will be recruited to the trial when there is maternal request in labour for opioid analgesia.

### Inclusion criteria

The women recruited will be those who have given written informed consent, who are in active labour with a singleton pregnancy, defined as regular uterine contractions of at least 2 in 10 minutes and cervical dilatation of at least 3 cms, with fetal gestational age of 37-42 weeks and a minimum weight of 60 kg. Competent women of sixteen years old or older are eligible. Both multiparous and nulliparous women will be eligible including those who have either gone into labour spontaneously or who have had an amniotomy and intravenous oxytocin to induce labour.

### Exclusion criteria

Exclusion criteria include lack of informed consent, allergy or previous severe reaction to opioid analgesia, opioid dependency, use of parenteral opioids within the previous 24 hours, history of fetal compromise, maternal cardiorespiratory compromise, ASA 3 & 4 (severe systemic disease including threat to life), maternal weight greater than 120 kgs or less than 60 kg.

### Allocation of analgesia

Once recruited, women will be randomly allocated to receive either intramuscular pethidine 150 mg or diamorphine 7.5 mg into the gluteus muscle or muscles of the lateral thigh. Qualassept Ltd is supplying the pre-filled syringes of pethidine and diamorphine to the respective pharmacies in Poole Hospital NHS Foundation Trust or the Royal United Hospitals Bath NHS Trust. The study drug will be dispensed in batches of two syringes by pharmacy. The syringes will be labelled only with a trial number in order to conceal group allocation from both the midwife and the parturient, and to ensure that if two doses are given, the same opioid is given both times. Randomisation will use random block sizes between 2 and 10 to ensure approximately equal group sizes, be stratified by centre and generated using PEPI [12]. The method is administered by the pharmacies and ensures parturients, researchers, maternity unit staff and trial statistician are blind to allocation. Parturients will also receive Metoclopramide 10 mg with the first dose, in line with current department prescribing guidelines. A maximum of two doses of opioid may be given with a minimum interval of 2 hours if the women requests additional analgesia. Regional analgesia or Entonox will be available as rescue analgesia.

## Measurements

• *General demographics*. Age, weight, gestational age, cervical dilatation at first request for analgesia, frequency of contractions, parity, spontaneous or induced labour, use of syntocinon, presentation, mode of delivery, drug administration to delivery interval. This information will be derived from the maternal notes and interview with the midwife taking care of the parturient.

• *Maternal Pain Evaluation*. Pain severity during last contraction will be assessed using a visual analogue score (VAS) (with anchor points of 0 = no pain at all and 10 = the most excruciating pain) and verbal scales of pain intensity during the last contraction (pain intensity score: 0 = no pain; 1 = mild pain; 2 = moderate pain; 3 = severe pain). In addition, the midwife will assess pain relief during the previous 30 minutes (pain relief score: 0 = none; 1 = slight; 2 = moderate; 3 = good, 4 = complete).

• *Maternal Sedation and Vomiting Evaluation*. Maternal sedation during the previous 30 minutes will be assessed by the midwife using sedation scores (0 = alert; 1 = mild i.e. occasionally drowsy and easily aroused; 2 = moderate sedation i.e. frequently drowsy but still easily aroused; 3 = severe sedation i.e. somnolent and difficult to rouse) and by measuring maternal oxygen saturation by pulse oximetry. Maternal pulse oximetry measurement is integral within the cardiotocograph (CTG) machine (Phillips PN/2703A) at the RUH and using the Masimo Rad 5 machine at PHFT. Vomiting will be assessed as a side effect with a yes/no answer pre-dose and during each subsequent thirty minutes period.

Maternal assessments will be made when the woman first requests analgesia and then every thirty minutes after the trial drug has been given, for a maximum of three hours or until delivery has occurred or additional analgesia is requested. A further 3 hours of measurements are then made if additional opioid analgesia is requested not less than 2 hours after the first dose. The parturient's overall satisfaction with the analgesia will also be assessed 12-24 hours post partum as well as their wish to choose the same opioid for their next labour analgesia.

• *Fetal Monitoring*. Fetal well-being will be monitored by continuous monitoring of fetal heart rate baseline and variability according to standard department protocols and classified as **Reassuring **(baseline 110-160 bpm, variability >5 bpm, no decelerations and accelerations present); **Non-reassuring **(baseline 100-109 or 161-180, variability of <5 for >40 but <90 minutes, and early or variable decelerations or a single prolonged deceleration up to 3 minutes) or **Abnormal **(Baseline <100 or >180 or sinusoidal pattern for >10 minutes, variability of <5 for >90 minutes, and atypical variable decelerations or late decelerations or a single prolonged deceleration of greater than 3 minutes). CTG monitoring is made using Corometrics 170 series machine in PHFT and using the Phillips PN/2703A machine at RUH. If meconium staining occurs then the time of first appearance in relation to administration of trial drug will be noted. At delivery cord blood will be taken for measurement of umbilical arterial and venous pH.

• *Neonatal Monitoring*. Neonatal condition will be assessed by time to first breath, Apgar score at 1 minute and 5 minutes, need for naloxone, need for resuscitation by bag mask ventilation or intubation and transfer to Neonatal Intensive Care Unit (NICU). Neonatal oxygen saturation will also be measured every 30 minutes from birth for 2 hours using the Masimo Rad 5 pulse oximeter at PHFT and Mindray PM-60 at RUH. Neonatal sedation in the first 2 hours of life will also be recorded using the same scoring system as the parturient. Time from delivery to first feed will be recorded as well as a subjective assessment of feeding behaviour by the midwife (0 = normal; 1 = mildly depressed rooting and sucking behaviour; 2 = severely depressed rooting and sucking behaviour) during the first 2 hours of life.

### Primary outcome measures

#### Maternal

##### Pain relief

Measured using change in pain intensity from pre-analgesia levels over the subsequent 3 hours.

##### Pain relief at 60 minutes

Measured by change from baseline in pain intensity VAS at 60 minutes. The dose of analgesia is administered intra-muscularly, and it is anticipated that maximum analgesic effect will occur at around 60 minutes after administration. Pain intensity at 60 minutes was the primary outcome used by Fairlie et al [10], and is the basis of our sample size calculation.

#### Neonatal

##### Side effects

(a) Need for neonatal resuscitation

(b) Apgar Score <7 at 1 minute

### Secondary outcome measures

#### Maternal

##### Pain

(a) Verbal pain intensity over first 3 hours

(b) Verbal pain intensity at 60 minutes post administration

(c) Pain relief score over first 3 hours

(d) Maternal satisfaction with analgesia 12-24 hours after delivery

##### Other

(e) Maternal sedation score over first 3 hours

(f) Time from first dose to delivery

(g) Entonox or regional analgesia given

(h) Nausea over 3 hours

(i) Vomiting over 3 hours

(j) Oxygen saturation over 3 hours after each dose of study drug or until delivery

(j) Percent reporting that they would choose the same pain relief in their next pregnancy

#### Neonatal

(a) Cardiotocography over the first 3 hours after each dose of study drug or until delivery

(b) Time from first dose to first meconium staining

(c) Umbilical artery pH and vein pH

(d) Time from delivery to first breath

(e) Apgar score at 5 mins

(f) Requirement for naloxone

(g) Neonatal oxygen saturation at 30 mins, 60 mins, 90 mins, 120 mins after delivery

(h) Time from delivery to first feed

(i) Feeding behaviour during first 2 hours

(j) Neonatal sedation in the first 2 hours after delivery

(k) Transfer to neonatal intensive care unit

## Sample Size

The number of women to be recruited has been determined using a sample size calculation, and is based upon the maternal primary outcome measure of pain relief at 60 minutes, and the neonatal primary outcome measures of Apgar score < 7 at 1 minute and neonatal resuscitation. We could find no data on our specific maternal primary outcome on which to base the sample size calculation (i.e. change in pain intensity). However, Fairlie et al in their randomised trial of pethidine versus diamorphine [10] reported on VAS pain intensity at 60 mins post administration of the first dose, and we have based our sample size calculation upon this. A standard deviation (SD) for pain intensity post opiate administration can be estimated from the data they present (SD = 2.6), and we have estimated the SD of change in pain intensity using 3 scenarios of different correlations between post-analgesia and pre-analgesia scores; correlations of 0.3, 0.5 and 0.7 give SDs of 3.1, 2.6 and 2.0 respectively. A 1 cm difference on a 10 cm VAS represents 10% of the scale, and to detect mean differences of this magnitude between the two arms of the trial with 90% power (at the 5% significance level) will require sample sizes of 203, 144 and 86 per group respectively in the 3 scenarios. A 1 cm difference is similar to the difference in pain intensity at 60 minutes found by Fairlie (0.9), is not larger than the 1.4 cm identified by Holdgate et al [13] as being the minimum change in pain that can be subjectively identified by patients with acute pain, and represents a standardised effect size of 0.38. We will set sample size at the higher figure of 203 per group.

Regarding neonatal outcomes, the Fairlie trial found that around 30% of neonates required resuscitation, and that around 25% of neonates whose mothers were given pethidine had 1 minute Apgar scores of less than 7 [10]. Thus with 203 participants per group the study will have 90% power to detect reductions of around 50% in both these outcomes (i.e 30% v 16% and 25% v 12% respectively). The difference for Apgar scores is similar to that found by Fairlie et al [10].

Initially we will aim to recruit 450 to allow for the fact that not all women will have data on pain intensity, for example if they delivered soon after administration of the trial drug. However we will monitor this during the trial and alter our recruitment target accordingly.

The recruitment target will be split evenly between the 2 trial sites. In Poole there are approximately 4000 deliveries per year and at Royal United Hospital Bath there are approximately 3,500 (based on 2008 figures). Assuming a low recruitment rate of 10%, and taking into account that pethidine is currently administered to approximately 40% of women, recruitment should take around 24 months.

## Statistical Analysis

Women will be analysed in the group to which they were originally randomised, regardless of what subsequently occurred during their labour. To enable this, where appropriate, all measures will be collected on all women once they have been randomised. However, within this framework, women could still, at any time, withdraw their consent to be in the trial.

Data will be analysed using SPSS for Windows and MLWin [14]. Significance tests will use a 5% two-sided significance level. Randomisation will be stratified by study site, and this will be controlled for in all analyses. Given the design of the trial, it is anticipated that missing data will be mostly negligible. The exception will be for the observations taken at 30 minute intervals after administration of the trial drug, where observations will cease if the mother delivers. We will assume these missing data are "missing at random" [15]. Analysis of the two maternal primary outcome measures will be as follows. Pain relief will be measured at 30 minute intervals over 3 hours. The repeated measures nature of the data will be taken into account using a maximum likelihood based multi-level (mixed) model [14]. This model permits analysis of unbalanced repeated measures data thus avoiding exclusion of participants with incomplete data [14]. Initially the interaction between trial group and timing of pain VAS measurement will be tested to see if the effects of the study drug change over time. If the interaction effect is not statistically significant, the interaction will be removed from the multi-level model and the main effect of trial group tested. Parameter estimates with 95% confidence intervals will be presented. The second maternal primary outcome measure, mean difference in pain relief at 60 minutes, will be estimated using multiple regression.

The neonatal primary outcomes, need for resuscitation and Apgar score under 7 at 1 minute, will be compared between groups using logistic regression, and the results presented as odds ratios (95% CI) and Numbers Needed to Treat (NNT).

Supplementary analyses will adjust for the following covariates that might have an impact on primary outcomes: maternal age, parity, gestation, pre-administration pain intensity. No formal correction for multiplicity will be applied to the significance tests for the 4 primary outcomes. Under the null hypothesis that the two treatment groups give identical outcomes, the probablities of 1 or more, 2 or more, 3 or more, or all 4 being statistcally significant are 0.19, 0.01,<0.001 and <0.001 respectively. The trial report will include a section on the impact of multiplicity on interpretation of the results.

Analysis of secondary outcome measures is likely to be as follows. The distributions of oxygen saturation and nausea VAS are likely to be highly skewed and so values will be categorised. For example, as under 95, 95-97, 98-100 for oxygen saturation and 0, 0.1-4.9, 5+ for nausea. These and other repeated measures categorical outcomes (verbal pain intensity, pain relief, maternal sedation, vomiting, cardiotocography) will be analysed in two ways; (a) using repeated measures models, and (b) as the proportion of women having a poor result during the 3 hour follow-up, using logistic regression (eg proportions having oxygen saturation 97 or below, nausea 5+, verbal pain intensity moderate or worse, pain relief none or slight, sedation moderate or severe, vomiting, cardiotocography non-reassuring or abnormal). Neonatal oxygen saturation and sedation scores in the 2 hours after delivery will be analysed using a similar approach.

Maternal satisfaction with analgesia and neonatal feeding behaviour (normal, mildly depressed, severely depressed) will be analysed using multinomial logistic regression.

Dichotomous outcomes such as Apgar score < 7 at 5 minutes, use of naloxone, transfer to neonatal intensive care unit, and reporting that they would choose the same analgesia for their next pregancy, will be analysed using logistic regression.

Time from first administration of study drug to delivery, and time to first meconium staining (excluding those where staining ocurred prior to trial drug), and time from delivery to first feed and time to first breath will be analysed using proportional hazards models, and the results presented as hazard ratios.

Multiple regression will be used to analyse umbilical artery pH and vein pH. Results will be presented with 95% confidence intervals whenever possible.

Both trial drugs are widely used in the NHS and internationally. No interim analysis is planned, but will be conducted if specifically requested by the trial Data and Safety Monitoring Committee. The main role of the DSMC will be to monitor serious adverse events.

Results will be reported using CONSORT guidelines [16].

## Retention of Data Records and Data Protection

Data records will be kept in a secure locked cupboard for a period of 30 years. All data will be held on RUH and PHFT computers, which are password protected. The data on computers will be anonymised and comply with the Data Protection Act.

## Adverse Event Reporting

### Definitions

An **Adverse Event (AE) **is defined as any untoward medical occurrence or experience in a patient or clinical investigation subject which occurs following the administration of the trial medication regardless of the dose or causal relationship. This can include any unfavourable and unintended signs or symptoms, an abnormal laboratory finding or a disease temporarily associated with the use of the protocol treatment whether or not related to the medicinal product. For example:

• any new diagnosis

• any symptom that requires medicinal clarification or leads to in-patient admission

• any suspected adverse drug reaction

• any symptom that appears on the participant's medical records

• any event related in time with the intake of the study medication and affecting the health of the participant (including laboratory value changes)

### Adverse Event Severity (AEs)

All AEs will be examined to determine severity. The intensity of an AE, as assessed by the investigator, will be recorded as either 'mild', 'moderate' or 'severe', as indicated by the following definitions:

• *Mild*: an event that requires minimal clinical treatment, or an AE requiring monitoring but no intervention or treatment; causes slight discomfort

• *Moderate*: an event that requires non-routine intervention; i.e. a new clinical treatment or diagnostic procedure, administered within an hour of the event; causes annoying discomfort.

• *Severe*: incapacitating with inability to do usual activities or significantly affects clinical status, and warrants intervention.

### Relationship of the Adverse Event to Study Treatment

An AE is considered associated with the use of the drug if the attribution is classified as "Possible", "Probable" or "Very Likely".

Causality definitions

**Unrelated**: There is sufficient information available to show that the aetiology is unrelated to the drug.

**Unlikely**: An AE for which an alternative explanation is more likely (e.g. concomitant medications, concomitant diseases), and/or the relation with time suggests that a causal relationship is unlikely.

**Possible**: An AE which might be due to the use of the drug. An alternative explanation (e.g. concomitant medications, concomitant diseases) is inconclusive. The relationship in time is reasonable; therefore the causal relationship cannot be excluded.

**Probable**: An AE which might be due to the use of the drug. The relationship in time is suggestive. An alternative explanation is less likely (e.g. concomitant medications, concomitant diseases).

**Very likely**: An AE, which is listed as a possible adverse reaction and cannot be reasonably, explained by an alternative explanation (e.g. concomitant medications, concomitant diseases). The relationship in time is very suggestive.

**Adverse Drug Reactions (ARs) **are responses to a drug which are noxious and unintended and which occur at doses normally used in man for prophylaxis, diagnosis, or therapy of diseases or for modification of physiological function. An adverse 'reaction', contrary to an 'event' is characterised by the fact that a causal relationship between the drug and the occurrence is suspected, i.e. judged possible by the reporting or a reviewing health professional.

An **Unexpected Adverse Drug Reaction **is any adverse reaction for which the nature or severity is not consistent with the applicable product information.

A **Serious Adverse Event (SAE) **is defined as any undesirable experience occurring to the patient, whether or not considered related to the protocol treatment. A **SAE **which is considered related to the protocol treatment is defined as a **Serious Adverse Drug Reaction (SADR).**

A **Suspected Unexpected Serious Adverse Reaction (SUSAR) **is classified as 'unexpected' i.e. a serious adverse reaction, the nature and severity of which is not consistent with the information about the medicinal product in question set out in the summary of product characteristics for the product.

Adverse events and adverse drug reactions which are considered as serious are those which result in death; a life threatening event; prolongation of hospitalisation; persistent or significant disability/incapacity, a congenital anomaly/birth defect; any other medically important condition (i.e. important adverse reactions that are not immediately life threatening or do not result in death or hospitalisation but may jeopardise the patient or may require intervention to prevent any of the other outcomes listed above).

### Reporting Procedure

The documentation of reporting of SAEs should be in accordance with ICH guidelines for Good Clinical Practice (5.17.1, 5.17.2 and 5.17.3) [17].

All serious adverse events (SAEs) or drug reactions (SDRs) (e.g. allergic reaction) arising from the use of the diamorphine or pethidine occurring from time of randomisation until 30 days after the last protocol treatment administration, will be reported to the following bodies:-

a) MHRA

b) Southampton and South West Hampshire Research Ethics Committee

c) Sponsor (Poole Hospital NHS Foundation Trust) via the local Trust adverse incident reporting form (AIRS form) and also to the Research Governance Departments of the respective Trusts.

d) Chair of the Data and Safety Monitoring Committee

Reporting will be done by fax within 24 hours of the initial observation of the event. The principal investigator will decide if these events are related to the protocol treatment or not. Completed documentation of any reported adverse events or serious adverse drug reactions will be returned within 10 days of the initial report by the principal investigator. SAE will be followed until significant changes return to baseline, the event stabilises or is no longer considered as clinically significant by the investigator, or patient dies or withdraws consent.

### Documentation of adverse events

Patients will be monitored for the duration of the study and any AEs occurring will be documented in their research files (CRFs).

The following information will be documented for each AE:

• nature of the event

• date and time of onset

• interval between the first administration of the study drug and the onset of the AE

• concomitant treatment; product, indication, dosage, dosage interval, presentation, mode of administration and administration regimen

• duration of the AE

• frequency

• severity (mild/moderate/severe)

• seriousness assessed according to the definition of an SAE as previously described

• causality assessed according to the causality definition previously described

• actions taken at the onset of AEs such as no measures, AE/symptom was treated medically, or other measures (specified)

• outcome, classified as resolved, unresolved, resolving, resolved with sequelae, death or unknown

• whether the event led to withdrawal from the study

## Steering Group

The Steering Group will comprise of the chief and principal investigators and co-applicants (anaesthetists, midwives, obstetrician, statistician) and will also consist of lay representative, a representative from the National Childbirth Trust, the Research Governance Manager and the Sponsor. The Steering Group will meet at least three times per year during the trial to discuss the conduct of the trial, recruitment targets, adverse events and any concerns arising from the trial.

## Data and Safety Monitoring Committee (DSMC)

The DSMC will consist of an independent chair, Prof. Philip Steer, Emeritus professor of Obstetrics, Imperial College, London; lay members, consultant anaesthetists, neonatologist and statistician with ethics experience all independent from the Steering Group. The chief investigator will provide the DSMC with minutes of the Steering Group meetings, anonymised details of all the adverse events and any other data requested by the chair of the DSMC.

## Discussion

This will be the first RCT with adequate power to compare the analgesic and side effect profiles of intramuscular diamorphine and pethidine for analgesia in labour. If diamorphine is demonstrated to be the superior drug in terms of efficacy and side effects, this could lead to a change in national practice in the United Kingdom to the benefit of mother, fetus and neonate based on good evidence. Pethidine is also widely used worldwide and this study has the potential to alter its worldwide usage.

## Intellectual Property Rights

We do not envisage any intellectual property rights issues.

## Abbreviations

VAS: visual analogue score; ASA: American Society of Anesthesiologists' classification of physical fitness; NICU: neonatal intensive care unit; CTG: cardiotocograph; SPSS: statistical package for the social sciences; CONSORT: consolidated standards of reporting trials; NHS – National Health Service.

## Competing interests

The authors declare that they have no competing interests.

## Authors' contributions

MW and JT conceived of the study, contributed to the design and co-ordination of the trial and the draft of the manuscript. MW is chief investigator and principal investigator at the sponsor site Poole Hospital NHS Foundation Trust. MW was responsible for applying to the Research for Patient Benefit (RfPB) programme for funding, obtaining ethics and MHRA approval for the trial and for providing regular reports to RfPB and the research ethics committee. MW is responsible for communication with the Data Safety Monitoring Committee and MW and JT jointly chair the Steering Group meetings. JT is the principal investigator at the RUH Bath site. PT is responsible for statistical design and analysis of the study and is a member of the Steering Group. SB will have overall responsibility for setting up the study and management of the project at RUH Bath as well as over-viewing the trial files at both sites, overseeing research assistants and recruitment. All authors have read and approved the final version of the protocol manuscript.

## Pre-publication history

The pre-publication history for this paper can be accessed here:

http://www.biomedcentral.com/1471-2393/11/51/prepub
